# Somatic genomic alterations in retinoblastoma beyond *RB1* are rare and limited to copy number changes

**DOI:** 10.1038/srep25264

**Published:** 2016-04-29

**Authors:** Irsan E. Kooi, Berber M. Mol, Maarten P. G. Massink, Najim Ameziane, Hanne Meijers-Heijboer, Charlotte J. Dommering, Saskia E. van Mil, Yne de Vries, Annemarie H. van der Hout, Gertjan J. L. Kaspers, Annette C. Moll, Hein te Riele, Jacqueline Cloos, Josephine C. Dorsman

**Affiliations:** 1 Department of Clinical Genetics, VU University Medical Center, Van der Boechorststraat 7, 1081BT, Amsterdam, The Netherlands; 2 Department of Medical Genetics, Center for Molecular Medicine, University Medical Center Utrecht, Universiteitsweg 100, 3508 AB, Utrecht, The Netherlands; 3 Department of Genetics, University Medical Centre Groningen, University of Groningen, 9700 RB, Groningen, The Netherlands; 4 Department of Pediatric Oncology/Hematology, VU University Medical Center, De Boelelaan 1117, 1081 HV, Amsterdam, The Netherlands; 5 Department of Ophthalmology, VU University Medical Center, de Boelelaan 1117, 1007 MB, Amsterdam, The Netherlands; 6 Division of Biological Stress Response, Netherlands Cancer Institute, Plesmanlaan 121, 1066 CX, Amsterdam, The Netherlands; 7 Department of Hematology, VU University Medical Center, de Boelelaan 1117, 1081 HV, Amsterdam, The Netherlands

## Abstract

Retinoblastoma is a rare childhood cancer initiated by *RB1* mutation or *MYCN* amplification, while additional alterations may be required for tumor development. However, the view on single nucleotide variants is very limited. To better understand oncogenesis, we determined the genomic landscape of retinoblastoma. We performed exome sequencing of 71 retinoblastomas and matched blood DNA. Next, we determined the presence of single nucleotide variants, copy number alterations and viruses. Aside from *RB1*, recurrent gene mutations were very rare. Only a limited fraction of tumors showed *BCOR* (7/71, 10%) or *CREBBP* alterations (3/71, 4%). No evidence was found for the presence of viruses. Instead, specific somatic copy number alterations were more common, particularly in patients diagnosed at later age. Recurrent alterations of chromosomal arms often involved less than one copy, also in highly pure tumor samples, suggesting within-tumor heterogeneity. Our results show that retinoblastoma is among the least mutated cancers and signify the extreme sensitivity of the childhood retina for *RB1* loss. We hypothesize that retinoblastomas arising later in retinal development benefit more from subclonal secondary alterations and therefore, these alterations are more selected for in these tumors. Targeted therapy based on these subclonal events might be insufficient for complete tumor control.

Retinoblastoma is a childhood cancer of the retina. Although the disease is relatively rare accounting for 2% of childhood cancers^1^, retinoblastoma is the most common intra-ocular malignancy in children^2^. From a clinical genetics perspective, three retinoblastoma types can be distinguished: familial (10%), sporadic heritable (30%) and non-heritable (60%). Patients with familial or sporadic heritable retinoblastoma have a germ line mono-allelic *RB1* mutation and have acquired a second *RB1* hit in the retina. While familial patients have inherited the mutant allele, sporadic heritable patients have acquired a *de novo RB1* mutation. The majority of non-heritable retinoblastoma (95%) is also caused by bi-allelic inactivation of *RB1* but in this case occurring through two subsequent somatic events in the developing retina. A minority of non-heritable retinoblastoma (2%) is caused by amplification of the oncogene *MYCN*^3^. Recently, chromothrypsis of chromosome 13 disrupting the *RB1* locus has been described as an alternative mechanism for *RB1* inactivation^4^. Possibly, 13q chromothrypsis accounts for the remaining patients for whom no *RB1* or *MYCN* alterations can be found by Sanger sequencing, Multiplex Ligation-dependent Probe Amplification (MLPA) or *RB1* promotor methylation assays.

Yet, while inactivation of *RB1* in the developing retina is sufficient for neoplastic onset, it has been suggested that additional genetic alterations are required for malignant progression^5^. In agreement, based on comprehensive genome-wide next-generation sequencing (NGS) efforts, it was claimed that two to eight genetic alterations are required to drive tumorigenesis^6^. Throughout the last decade, useful insights about secondary genetic alterations in retinoblastoma have been obtained by studies profiling large (>50 Kb) somatic copy number alterations (SCNAs). Unlike many other cancers, very little is known about smaller genetic alterations (<50 Kb) that are typically identified by genome-wide NGS. To date, only 14 retinoblastoma samples have been profiled for single nucleotide variations (SNVs) and insertions and deletions (INDELs) in a genome-wide fashion^7^,^8^.

Our current study set out to determine the prevalence of SNV and INDELs in order to facilitate the identification of the genetic alterations that drive retinoblastoma development. To this end, whole-exome sequencing (WES) of a diverse set of retinoblastoma and matching blood DNA was performed (N = 71). Using dedicated bioinformatics and statistics, this dataset allowed for the identification of SNVs/INDELs, SCNAs including chromothrypsis, loss of heterozygosity (LOH) and viral content. By integrating genomics with clinical and histopathological data, our study aims to provide a detailed molecular landscape, which may help better understand retinoblastoma development.

## Results

### Identification of significantly mutated genes by somatic SNV/INDEL analysis

To identify genes relevant for retinoblastoma carcinogenesis, we compared SNVs/INDELs observed in 71 primary retinoblastoma samples with a variant database established from blood DNA of the same patients (N = 70, from one bilateral patient, one tumor sample per eye was analyzed). Sequencing, alignment and enrichment statistics are provided (Table S1). The number of exonic variants ranged between 18,510 and 23,772 variants per tumor sample, adding up to a total of 1,386,285 variants in the tumor cohort. By comparing tumor variants with the pooled blood variant database, only 5,797/1,386,285 (0.41%) variants were considered somatically acquired. Using the criteria for possible pathogenic variants (see Materials and methods, chapter 1.4), only 258/5,797 (4.45%) of the somatic variants remained (Table S2). These 258 somatic variants were distributed over 202 unique genes, of which only 3 genes were recurrently (i.e. at least two different patients) mutated; *RB1* (51 variants, 44 patients), *BCOR* (5 variants, 5 patients), and *CREBBP* (2 variants, 2 patients). For tumors with SNVs/INDELs in *RB1*, tumor cellularity could be estimated based on the variant allele frequency (VAF). Patients that were compound heterozygous for *RB1* (confirmed by conventional DNA diagnostics) had a mean VAF close to 0.5 (mean 0.48) and patients with homozygous *RB1* mutations (confirmed by conventional DNA diagnostics) had a mean VAF close to 1 (mean 0.98), indicating high tumor cellularity in our cohort. Variant details for *BCOR* and *CREBBP* genes are given in Table 1. VAFs of *BCOR* and *CREBBP* were well below 1, indicating that not all alleles were mutated and therefore possibly not all tumor cells harbored these secondary variants. In short, although relatively lenient thresholds were used for pathogenicity and recurrence, aside from *RB1*, only two genes were identified as recurrently hit by somatic and possibly pathogenic mutations.

In parallel to our custom defined filtering strategy MutSigCV^9^ was used, a tool purposed to find significantly mutated genes based on frequency and adjusting for sequence content and coverage. Somatic variants that were not located in segmental duplication regions, had at least 10x sequencing depth and were flagged “PASS” by the GATK variant filter were used as input for MutSigCV (2085 variants). Only *RB1* was significantly mutated (q-value 3.35E-11) (Table S3). Although *BCOR* was not considered significantly mutated after multiple testing correction (p-value 1.30E-03, q-value 1), notably this gene ranked sixth in the MutSigCV gene list.

### Somatic copy number alterations

**Figure 1:**
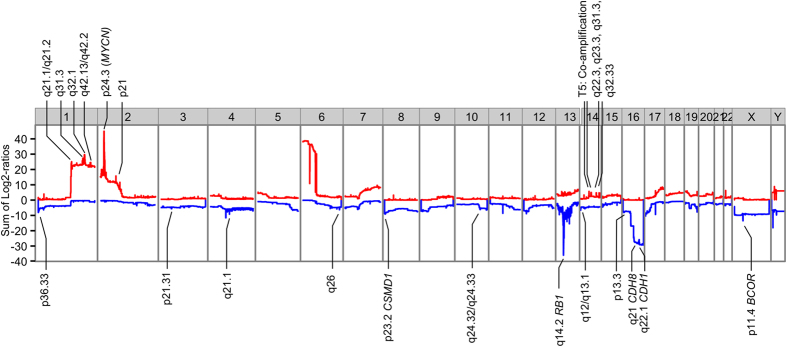
Significantly altered copy number regions identified by GISTIC. A karyogram overview of the cumulative copy number changes (Log2-ratios of tumors versus germ line summed over 71 samples, Y-axis) is shown separately for gains (red) and losses (blue). Based on SCNA focality, amplitude and recurrence, GISTIC identified significantly altered regions, which are highlighted by cytoband labels. For regions that contained a single gene only, the gene symbol is given.

Although WES was primarily designed for the identification of exonic SNVs/INDELs, WES data can also serve well for copy number alteration profiling. Using off-target sequencing reads that are not subject to systematic biases due to bait hybridization efficiencies, we performed SCNA analysis implemented by CopywriteR^10^. Segmented results are available (Table S4) and when the file suffix is renamed to .seg it can be used for convenient browsing of the SCNA results in Integrative Genome Viewer (IGV)^11^. For each HGNC approved gene, the sum of Log2-ratios (calculated based on normalized read depth in tumor divided by matched germ line) is plotted along genomic coordinates (Fig. 1). The sum of Log2-ratios is proportional to the total number of acquired DNA copies in all tumor samples (N = 71) for the respective gene. To identify significantly altered genes, which appear as spikes in Fig. 1, GISTIC analysis was performed. Highlighted cytoband labels in Fig. 1 indicate regions identified by GISTIC as significantly altered. Putative target genes are highlighted only if a single protein-coding gene was located at the identified region. All significantly altered regions are given including RefSeq gene annotation (Table S5). One of the significantly altered regions was located at Xp11.4 and included *BCOR* exclusively. Instead of SNVs, two tumors (T22 and T67) showed focal homozygous loss of *BCOR* adding up to a mutations frequency of 7/71 (10%) tumors.

**Figure 2:**
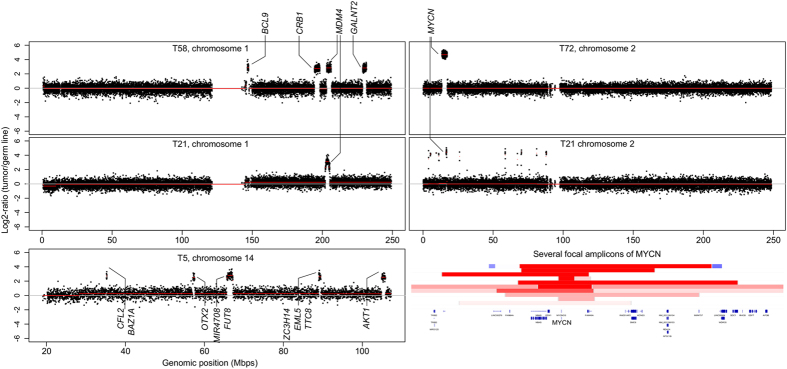
Examples of high-level gains. Segmented (orange lines) somatic copy number estimates (black dots, Log2-ratios, Y-axis) are plotted along genomic coordinates (X-axis). Focal and high-level gains were scarce and were restricted to chromosomes 1 (2/71 tumors), 2 (6/71) and 14 (1/71). Established oncogenes contained in the focal gains are highlighted. Only for chromosome 2, the intersection of amplicons included a single gene only (*MYCN*). Tumors T58, T21, and T5 showed co-amplification of multiple loci at chromosome 1, 2, and 14 respectively.

High-level amplifications (in our study defined by >10 copies, Log2-ratio >2.32) were observed at 1q, 2p and 14q and are visualized in more detail in Fig. 2. Amplifications of the four regions at 1q were divided over two tumors, one tumor (T58) with similar copy number for all four regions indicating co-amplification and one tumor (T21) with only 1q32.1 amplification including *MDM4* and 26 other genes (Table S5). High-level amplification of 2p24.3 (*MYCN*) occurred in 6/72 (8%) tumors of which 4/6 (67%) lacked mutations in *RB1* (*RB1*^−/−^*MYCN*^*A*^)^3^. The tumor with focal 1q32.1 amplification (T21) also showed focal *MYCN*-amplification and several additional focal amplifications at 2p with similar copy numbers, indicating co-amplification. One previously described tumor^12^ had high-level amplification of five focal amplifications at 14q with similar copy numbers as well (Log2-ratio 2.75, ploidy 2^*^2^2.75^ =  13.5 copies). All high-level focal amplicons showed LOH, indicating that only one of the two alleles was amplified (haplotype amplification) consistent with earlier reports of *MYCN* amplification in neuroblastoma^13^.

**Figure 3:**
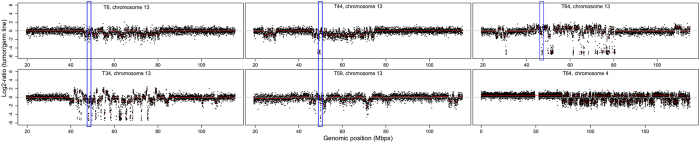
Chromothrypsis of chromosome 4 (1/71 tumors) and 13 (5/71 tumors). Segmented (orange lines) somatic copy number estimates (black dots, Log2-ratios, Y-axis) are plotted along genomic coordinates (X-axis). Chromothrypsis, characterized by clustered chromosomal alterations, was observed for 6 chromosomes in five different tumors. One tumor (T64) showed chromothrypsis at both chromosome 4 and 13. Blue rectangles indicate the *RB1* locus.

Six cases of chromothrypsis, defined as (sub-) chromosomal shattering, were observed in five different tumors (T6, T34, T44, T59, T64, Fig. 3) for chromosome arm 13q (5/6) and 4q (1/6). For each of the five 13q regions showing chromothrypsis, the *RB1* allele was included, indicated by the blue rectangles in Fig. 3. For 2/5 tumors with chromothrypsis at 13q, SCNAs were all losses consistent with initial reports about chromothrypsis^14^ while for 3/5 tumors SCNAs were both gains and losses. Tumor T64 had chromothrypsis of both 13q and 4q.

**Figure 4:**
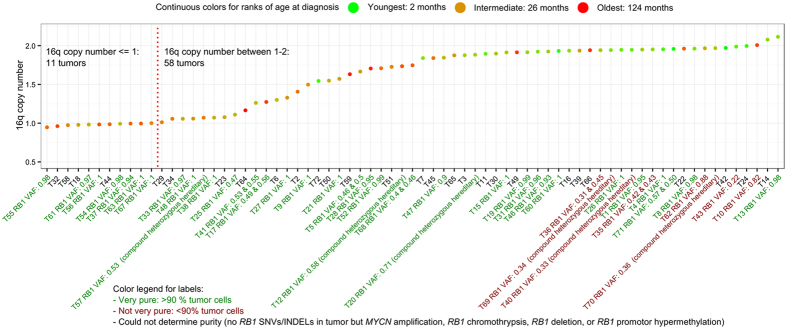
Amplitudes of 16q loss indicate tumor clonality. For each tumor (dots) the copy number of 16q is plotted, ordered by increasing copy number. A green-to-red color scale was mapped to age at diagnosis, showing a significant positive association between age at diagnosis and 16q loss amplitude (Kendall’s rank correlation test, p-value 1.2E-06). The tumor labels included VAFs of *RB1* variants from which contamination with non-tumor cells can be inferred. Although the majority of tumors were considered very pure (>90%, green labels), 16q loss did rarely reach change of one copy (11 tumors with ploidy <=1), suggesting of within-tumor heterogeneity.

The most frequent large-scale SCNAs were gain of 1q, 2p, and 6p and loss of 16q, in agreement with published studies^12^,^15^,^16^,^17^,^18^,^19^,^20^,^21^. This SCNA profile is typical for retinoblastoma and is not observed in other cancers^22^, indicating that these alterations may confer a growth advantage for retinoblastoma lineages specifically. For the majority of 1p, 2p, 6p, and 16q alterations (91/132, 69%, Table S6), the SCNA did not exceed change of one copy. For illustration, copy numbers of 16q are visualized for each tumor (Fig. 4). 69/71 (97%) of the tumors showed 16q copy number less than two, although only 11/71 (15%) showed copy number below one. VAFs of *RB1* are included in the labels and indicate the high tumor cellularity for the majority of the cohort, meaning contamination of non-cancer cells cannot explain incomplete loss of 16q. The remaining explanation is that not all tumor cells showed 16q loss indicating within-tumor heterogeneity for the majority of the cohort. The amplitude of 16q loss may reflect the fraction of cells with 16q loss.

**Figure 5:**
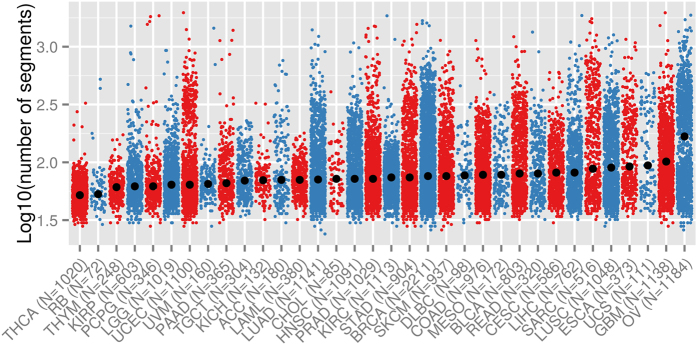
Number of SCNAs across different cancer types. For each tumor (dots) the number of contiguous DNA segments (so wild type is 22 + X + Y = 24 segments, log_10_(24) = 0.38) is plotted for our retinoblastoma cohort and all cancer types available at TCGA in alternating colors. Cancer types are ordered by increasing median of SCNAs, showing that the retinoblastoma genome has relatively few SCNAs compared with cancers available in TCGA.

By comparing SCNAs of 46 retinoblastoma samples made by single nucleotide polymorphism (SNP) arrays with 153 high-grade serous ovarian cancer, it was claimed that retinoblastoma genomes are remarkably stable^20^. To compare the level of SCNAs of retinoblastoma with other cancer types, the total level of genomic disturbance of our cohort was compared with all tumor samples available at The Cancer Genome Atlas (TCGA, N = 22,455 tumors)^23^. For each individual tumor the total genomic disturbance, represented by the number of DNA segments with equal copy number, is plotted per tumor type (Fig. 5). The tumor types are ordered by the median of total genomic disturbance, confirming that retinoblastoma genomes attained relatively few SCNAs compared to other cancers. It is unclear whether this is a characteristic of pediatric cancer as no cancers types that exclusively occur in children are covered in the TCGA data set.

### Validation of identified SNVs/INDELs and SCNAs by *RB1*

To demonstrate the sensitivity and specificity of the used sequencing methodology for somatic and pathogenic variant detection, WES was compared with standard molecular *RB1* diagnostics based on Sanger sequencing and multiplex ligation-dependent probe amplification analysis. For heredity determination, blood DNA diagnostics was performed for all patients, except for T29 (foreign patient). For a subset of patients (36/71, 44%), tumor *RB1* diagnostics was performed as well, to aid diagnosis of retinoblastoma heredity. Using a combination of previously described SNV/INDEL and SCNA analysis, the primary disease causing genetic event (*RB1* or *MYCN* alterations) was determined based on WES data and compared with conventional *RB1* diagnostics (Supplementary Fig. 1).

For 59/71 (83%) tumors, *RB1* inactivation (either by SNV/INDEL or SCNA detection) or high-level focal amplification of *MYCN* was detected by WES (WES positives). In 31/59 (53%) of WES positives, conventional tumor *RB1* diagnostics was performed and the mutation detected by WES was exactly the same as identified by conventional diagnostics, indicating a high true positivity rate. For 28/59 (47%) of WES positives, no tumor DNA diagnostics was available. However, for 6 WES positives without tumor DNA diagnostics, a germ line mutation in *RB1* was identified that was always consistent with the somatic *RB1* mutation detected by WES, again indicating a high true positivity rate. However, for tumor T7 an *RB1* variant was detected by germ line DNA diagnostics in exon that was not detected by WES in the tumor (high GC content, poor coverage), although WES data showed LOH at the *RB1* allele. Since, tumor T7 had acquired focal high-level gain of *MYCN,* based on WES data this amplification would have been falsely considered the primary event.

For 12/71 (17%) tumor samples, no *RB1* mutations or *MYCN* amplification could be found by WES (WES negatives). For 4/12 WES negatives, tumor DNA diagnostics was performed. For one tumor (T16), indeed no second *RB1* hit or *MYCN* amplification was found by conventional DNA diagnostics (true negative). For the other 3 WES negatives, *RB1* mutations were found by conventional diagnostics including a tumor with promotor hypermethylation followed by LOH, one tumor with deletion of the promotor region and subsequent LOH and one tumor with compound heterozygous splice-site INDELs that were not annotated by Annovar in the WES-data. For the remaining 8/12 WES negatives, no tumor DNA diagnostics was performed but for 6 of them, a germ line mutation was found. All these 6 patients that were WES negative but had a germ line *RB1* mutation showed clear LOH at the *RB1* allele in the WES data. Apparently, somatic mutations of *RB1* were missed by WES for these 6 tumors. One patient had a pathogenic deep intronic mutation (intron 18 inversion and splice site mutation), three had complicated insertions and two had mutations that were missed by WES because of thresholds for calling variants homozygous (*RB1* variant in T39 VAF 81% and in T45 VAF 78%) and therefore somatic.

In short, all *RB1* variants identified by WES that passed criteria to be called somatic and pathogenic were confirmed by conventional methods. Assuming that all tumors in our cohort either had *RB1*-inactivation or *MYCN* amplification, the sensitivity of WES was 83% (59/71). Incomplete sensitivity can be explained by epigenetic events, complex insertions, variants outside WES target areas and used thresholds for calling variants somatic.

### Quantification of viral traces in retinoblastoma tumor and blood

After an HIV outbreak in Zambia, the incidence of retinoblastoma was increased, suggesting a role for oncoviruses in retinoblastoma development^24^. It was also shown that the use of barrier methods of contraception, associated with decreased risk for human papilloma virus (HPV) infection, significantly decreased the risk of having a child affected by retinoblastoma^25^. Since HPV is known to inhibit the *RB1* pathway^26^,^27^,^28^, HPV infection is a plausible driver for retinoblastoma development alternative to - or in concert with *RB1* mutations. However, controversies exist surrounding the presence of viruses in retinoblastoma tissue^29^,^30^. To test whether viral DNA was present in our tumor cohort, we aligned all reads to all available viral reference genomes (2547 viruses) and counted the number of well-mapped reads per virus. As a positive control for the detection of viruses in our WES data cohort, the Enterobacteria phage phiX174 sensu lato (phiX174) virus was used. The phiX174 virus DNA was spiked into sequencing libraries to guarantee enough library complexity required for proper Illumina sequencing. Virtually all reads that mapped to viral reference genomes (99.8%) mapped to phiX174. The remaining viral reads (0.2%) mapped to 26 viruses, which have not been associated with cancer previously (Table S7). In all, no evidence could be found for the presence of tumor viruses in our retinoblastoma cohort.

### Testing for mutual exclusivity of identified somatic events and correlation with phenotype data

The identification of negative correlations between genetic events can give important insights into the molecular signaling pathways that underlie carcinogenesis. For example, the *RB1* pathway is inactivated in all glioblastoma tumors but is caused either by somatic *RB1* mutations, *CDKN2A/B* mutations, or *CDK4* amplification^31^. The power of analyses aimed to identify negative correlations depends on the overall sample size and the frequency of occurrence of the event pairs. To be more specific, if two events rarely occur, a relatively big sample size is required to detect a significant negative correlation. Since retinoblastoma is a rare disease and identified genetic events beyond *RB1* inactivation appear to be rare except for large SCNAs, identification of mutually exclusive events might be challenging.

**Figure 6:**
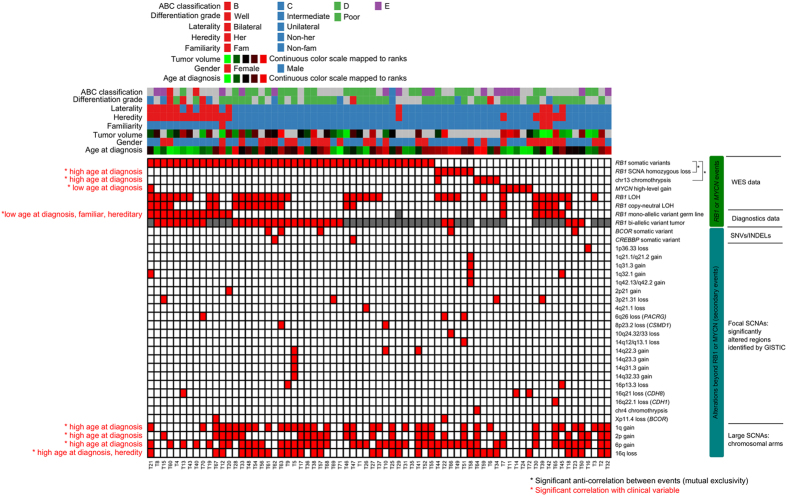
Landscape of somatic alterations in retinoblastoma. A binary event matrix (red: event occurred, white: event did not occur, grey: data not available) for SNVs/INDELs and SCNA, aligned with color-coded sample information. Columns represent tumor samples and rows represent events. For focal SCNAs containing a single gene only, the gene symbol is given in parentheses. Events that showed significant negative correlation are paired with black lines highlighted on the right of the matrix. Significant correlations between events and clinical variables are indicated by red labels on the left of the matrix. For large SCNAs (1p, 2p, 6p, and 16q), tumors with a mean ploidy more than 2.3 or less than 1.7 were called altered (red boxes).

Nevertheless, to identify negative correlations and ultimately signaling pathways, a binary event matrix was compiled from the identified SNV/INDEL and SCNA analysis (Fig. 6). For each pair-wise combination of somatic events, the phi correlation coefficient was calculated and tested for significance. After correcting for multiple hypothesis testing, significant negative correlations were only found between *RB1* SNVs/INDELs with either *RB1* loss (FDR = 0.01) or chromothrypsis at chromosome 13 (FDR = 0.03). High-level focal amplification of *MYCN* and *RB1* mutations by was not considered significantly inversely correlated because tumor T21 acquired both alterations (single nucleotide frameshift deletion in exon 15 of *RB1*; NM_000321:c.1397delA:p.E466fs). Also for tumor T7, from a non-familial unilateral patient, both high-level amplification of *MYCN* and *RB1* inactivation was observed (20 nucleotides frameshift insertion in exon 1; NM_000321:c.25ins20:pT9fs), although *RB1* inactivation was missed by WES because of low exon 1 coverage. However, LOH at the *RB1* locus was clearly identified through WES which is a strong indication of *RB1* inactivation.

Each event in the binary event matrix was tested for statistical association with the phenotypic variables aligned on top of the event matrix (Fig. 6). The phenotypic variable that correlated with most of the events was age at diagnosis. While focal high-level *MYCN* amplification and *RB1* germ line mutations by DNA diagnostics were associated with young age at diagnosis, homozygous *RB1* loss, chromothrypsis of chromosome 13, and alterations of chromosomal arms were associated with old age at diagnosis. By definition, *RB1* germ line mutations detected by DNA diagnostics were associated with familial and heritable retinoblastoma. No significant relations between SNVs/INDELs of *BCOR* or *CREBBP* with clinical variables were found.

## Discussion

The introduction of NGS enabled researchers to study genetics at unprecedented speed and dramatically reduced costs^32^. In particular, cancer research has benefited from the NGS development and as a result, genetic defects underlying many cancer types were revealed^33^. However, retinoblastoma is largely underrepresented in whole-exome/genome sequencing efforts with only 14 retinoblastoma samples profiled to date^4^,^20^. Our study is the first WES study that determined the somatic genomic landscape of retinoblastoma. Considering the low incidence of retinoblastoma, the sample size of our study was relatively large and therefore enabled powerful analyses.

Genes recurrently hit by SNVs/INDELs aside from *RB1* were restricted to *BCOR* and *CREBBP* and were only observed in the minority (5/71 (7%) and 2/71 (3%) tumors respectively) of tumors. Somatic pathogenic *BCOR* mutations were reported in retinoblastoma before with similar frequency (6/46 tumors, 13%)^20^. For the *BCOR* gene, next to 4 truncating (two stop-gain mutations, two frameshift deletions) and one non-synonymous SNV, homozygous somatic copy number losses were observed in two tumors (T22, and T67). This indicates that the observed *BCOR* mutations are loss-of-function mutations, in agreement with reports about deleterious *BCOR* mutations in acute myeloid leukemia^34^. Germ line mutations in *BCOR* can give rise to two X-linked syndromes characterized by microphthalmia^49^ and other malformations: Lenz microphthalmia and oculofaciocardiodental (OFCD) syndrome^35^, characterized by retarded eye growth. The function of *BCOR* appears to be crucial during early development, since knock-down of *BCOR* expression during embryogenesis in drosophila caused severe developmental perturbations of the eye, skeleton and central nervous system. Given that *BCOR* gene function is associated with proper eye development, it seems plausible that inactivation of this gene can be related to retinoblastoma.

For an even smaller fraction of our cohort (2/72, 3% tumors), *CREBBP* mutations were found. Heterozygous germ line mutations in *CREBBP* are known to cause the Rubinstein-Taybi syndrome (RSTS)^36^, characterized by mental retardation, growth retardation and distinct dysmorphology. There have been several reports of RTST patients with rare pediatric neural tumors^37^. Analysis of *CREBBP* mutations in RSTS patients showed that the mutations were concentrated (44.7% of RSTS patients) in the histone acyltransferase (HAT) domain (295/2442 (12%) of amino acids make up the HAT domain in *CREBBP*)^38^, indicating that the HAT domain is crucial for proper *CREBBP* function. The somatic *CREBBP* mutation in sample T62 is located in the most highly conserved (11 organisms, up to frog) region of the HAT domain. The *CREBBP* mutation in tumor T47 was not located in any of the *CREBBP* protein domains. Assuming *CREBBP* mutations were heterozygous (expected VAF 0.5) similarly to RSTS patients, given the VAFs (0.17 and 0.27) a quarter to about half of the cells were mutated for *CREBBP*. In addition to SNVs, tumor T48 had a heterozygous focal SCNA loss at 16p13.3 covering *CREBBP* exclusively. Focal SCNA losses of *CREBBP* in retinoblastoma tissue were also recognized previously at low frequency (2/94, 2% tumors)^4^. Since this event is so rare and amplitudes of loss are not very high (1/72, 1%), GISTIC analysis (Fig. 1, Table S5) did not consider this locus as significantly altered.

Apart from the three genes (*RB1*, *BCOR*, *CREBBP*) that showed somatic mutations in at least 2 different tumors, there were 199 other genes that were mutated in a single tumor only. Since these genes were mutated so infrequently, the molecular and clinical significance of these genes for retinoblastoma carcinogenesis is not apparent. Yet, in case these genes comprise different key components of a particular molecular signaling pathway, they could be very valuable for understanding retinoblastoma biology. Enrichment analysis of gene ontologies, including biological processes, pathways, diseases and many more, did not result in any significantly overrepresented ontology. This doesn’t necessarily mean all infrequently mutated genes are irrelevant but means no coherence could be found and therefore their relevance could not be demonstrated. Possibly, this gene list is polluted with false positives that are introduced due to artifacts in local INDEL realignment (GATK) PCR, sequencing, mapping and/or stochastic processes that influenced genotype calling. The possibility of false positive variants that can randomly affect genes signifies the importance to focus on recurrently mutated genes.

The number of SCNAs in our retinoblastoma samples was relatively low compared with other cancer types from the TCGA data set. Since loss of *RB1* under certain conditions may lead to genomic instability through missegregation of chromosomes eventually causing aneuploidy^39^,^40^,^41^,^42^, the relatively few SCNAs might appear surprising. Although our data showed that retinoblastoma is the second least disrupted cancer out of all TCGA cancer types, it is of note that no pediatric cancer types are covered in the TCGA data set. It is known that pediatric tumors have lower mutation rates than adult tumors^6^ and therefore, comparison with other pediatric cancers like neuroblastoma (unfortunately unavailable in TCGA) might provide another perspective. Also, although the genomic fraction hit by SCNAs in retinoblastoma might be relatively low compared with adult tumors, SCNAs in retinoblastoma were concentrated at specific genomic regions indicating their significance. For example, gain of chromosome arm 6p was observed in the majority of tumors (48/71, 68%) and only the minority (12/71, 17%) did not show any SCNA at 1p, 2p, 6p or 16q. Although most of the samples had little non-cancer contamination, losses of 16q often did not exceed loss of one copy, indicating that 16q loss is subclonal. Similarly, VAFs of *BCOR* and *CREBBP* and amplitudes of most of the large SCNAs were consistent with intra-tumor heterogeneity.

Although SCNAs were not detected in all tumors, we hypothesize that all tumors were aneuploid although with varying degrees. For example, in 58/69 (84%) tumor samples with 16q copy number decrease, the 16q copy number was between one and two copies, even in samples with very high tumor cellularity. We hypothesize that previously described *RB1* loss induced genomic instability can affect any chromosome but selection pressure of growth favoring SCNAs is required for SCNAs to exceed our detection thresholds. Since SCNAs were less abundant in tumors of patients diagnosed at young age, we hypothesize that clonal selection was less persevere in these patients. Possibly, tumors that arose during early stages of retina development originated from more undifferentiated precursors. The intrinsic proliferative capacity of these cells probably was sufficient for development of full-blown tumors and therefore there was no window for clonal selection on SCNAs. Lesions that arose at later stages of retinal development might have been neoplastic, but specific SCNAs favored growth of these cells and therefore cells with favorable SCNAs were selected for.

The significance of regions and genes hit by SCNAs was determined by GISTIC which makes use of SCNA focality, amplitude and recurrence. For few GISTIC regions, a single gene was included (e.g. *MYCN*, *RB1*, *BCOR*), while most GISTIC regions harbored multiple genes complicating target gene identification. Focal gains at chromosome 1q in human retinoblastoma are rare but in murine retinoblastoma, focal gains of *MDM2* were reported^43^. Human paralog *MDM4* has been proposed as putative retinoblastoma driver^5^,^44^ and was one of the 27 genes included in the significantly altered 1q31.2 region (Figs 1 and 2, Table S5). By inhibiting p53-induced apoptosis, gain of *MDM2/4* is suggested to prevent major cell death in *RB1* inactivated retina^45^. However, cone photoreceptor precursor cells, the alleged retinoblastoma cells of origin, show intrinsic p53-inhibition by high expression of *MDM2* that might leave gain of *MDM2/4* redundant. Furthermore, since *TP53* mutations in retinoblastoma never have been found, inactivation of p53 seems not to be a requirement for retinoblastoma development. Opposed to glioma where minimal regions of 1q gain exclusively included *MDM4*, target gene identification of 1q gain in retinoblastoma is not conclusive yet. Possibly, focal or single gene gains at 1q are rare because multiple genes at 1q are relevant for retinoblastoma development, illustrated by tumor T21 that showed focal gains at four separate 1q regions. Similarly, focal alterations for 6p in retinoblastoma were not found in our cohort, indicating multiple 6p loci could be relevant for retinoblastoma carcinogenesis. Although *E2F3* has been presented as 6p candidate gene^18^,^46^, the neighboring gene *SOX4* was gained with similar frequency in our cohort and was the exclusively gained gene in urothelial carcinoma^47^. Also for 16q loss, identification of the target gene is not completely conclusive. In our cohort, two GISTIC regions at 16q were identified exclusively including *CDH1* and *CDH8* respectively. However, *CDH11* which is located in between *CDH1* and *CDH8* was identified as the most frequently lost gene of 16q previously^48^. It was shown that inactivation of *CDH11* in murine retinoblastoma accelerated tumor growth validating its retinoblastoma suppressive function^49^. However, this does not directly discriminate *CDH1* and *CDH8* as retinoblastoma suppressors, and these discrepancies signify the difficulty of pinpointing a single gene as driver. We conclude that, unless the intersection of SCNAs exclusively covers a single gene like for *RB1*, *MYCN* or *BCOR*, identification of SCNA target genes remains challenging.

This study aimed to identify recurrent genetic alterations subsequent to *RB1* loss that drive tumor progression. Targeted disruption of gain-of-function alterations that are crucial for retinoblastoma cells to survive may lead to cell death. Our analyses of enucleated retinoblastoma showed that SNVs/INDELs beyond *RB1* inactivation were extremely rare. Also, since the VAFs of the secondary variants were well below 1, these variants most likely were not present in all tumor cells. Therefore, targeted treatment based on these secondary alterations, possibly can only disrupt parts of the tumor in parts of the retinoblastoma population. For several tumors, inactivation of *RB1* alone was sufficient to develop tumors that were so advanced they had to be enucleated. In agreement with the drastic consequences of *RB1* loss during early childhood, only 1% of the children who carry an *RB1* mutation remain unaffected^50^, indicating not much additional factors are involved. Given the high penetrance and the requirement for bi-allelic *RB1* inactivation, the second hit almost inevitably occurs. Possibly, the high chance of acquiring a second *RB1* inactivation can be explained by *RB1* haploinsufficiency. It was shown that heterozygous inactivation of *RB1* in RPE cells resulted in increased incidence of chromosome missegregation during mitosis^51^. Possibly, heterozygous *RB1* inactivation increases the risk for (copy neutral) LOH and partly explains the high disease penetrance.

In contrast to SNVs/INDELs, larger genetic alterations concentrated at specific genomic regions were more common, in particular for patients diagnosed at a later age. Only when *RB1* inactivation occurred at later age, additional genetic alterations might have had sufficient proliferative value to get selected for. Although SCNAs might be relevant for tumor growth in at least a subset of the retinoblastoma population, the clinical relevance remains to be determined. Furthermore, SCNAs might be associated with increased tumor progression but inhibition of these genetic events does not necessarily reverts the phenotype. Also, similarly to secondary SVNs/INDELs, the majority of SCNAs were subclonal and therefore targeting these alterations specifically might be insufficient for complete control of retinoblastoma.

## Materials and Methods

### Tissue collection

Tumor samples were obtained from retinoblastoma patients after primary enucleation and peripheral blood samples were collected at initial presentation before treatment. Tumor samples were snap frozen in liquid nitrogen and stored at −80 °C until further analysis. All patient samples and clinical and histopathological features were collected and stored according to local ethical regulations. All patients gave consent verbally, as this was the standard in the time the included patients were diagnosed. Since genetic analyses of our study focused on tumor DNA and not germ line DNA, waiver of informed consent was specifically given for genetic analyses by The Medical Ethics Review Committee of the VU University Medical Center which is registered with the USA OHRP as IRB00002991. The FWA number assigned to VU University Medical Center is FWA00017598. It was required that germ line variants were anonymized and pooled during somatic small variant identification. All experimental protocols used in this study were approved by the Medical Ethics Review Committee of the VU University Medical Center and all methods were carried out in accordance with the approved guidelines.

### Sample preparation for whole-exome sequencing

Genomic DNA from frozen tumor retinoblastoma specimens (N = 72) and matching white blood cell DNA (N = 71, 1 bilateral patient: one tumor sample per affected eye, 1 blood sample) was isolated with the NucleoSpin Tissue kit (Macherey-Nagel, Düren, Germany) or Wizard Genomic DNA Purification Kit (Promega, Madison, USA). DNA quality was analyzed for high molecular bands >20 kb by agarose gel electrophoresis. DNA concentrations were determined by Qubit 3.0 Fluorometer (Life technologies, Bleiswijk, The Netherlands). DNA yields and quality were within the same range for all samples. Genomic DNA was sheared using Covaris Focused-ultrasonicator (Covaris, Woburn, USA). Quality control of fragmented DNA was performed with Genomic DNA ScreenTape (Agilent technologies, Santa Clara, USA). DNA end-repair and ligation of sequencing and indexing adapters was done using Truseq Nano DNA library prep kit (Illumina, San Diego, USA). Exon enrichment was performed using SeqCapEZ v3.0 (Roche Nimblegen, Madison, USA). Sample preparation was performed in two batches, the first batch comprised tumor samples T1-T24, the second batch T25-T72. Within batches, tumor IDs were randomly assigned. Six samples were pooled per sequencing library and sequenced with 125 bps paired-end sequencing, HiSeq 2500 HT v3/4 (Illumina) yielding on average 40.6 million read pairs (range 28.6–56.8 million read pairs, N = 143).

### Pre-processing of sequencing reads and quality control

Adapter removal and 5′-end quality trimming was performed using Trimmomatic^52^ using default parameters. Read quality control of cleaned data was done with FastQC. Clean sequencing reads were mapped to UCSC genome version hg19 with Burrows-Wheeler aligner (BWA)^53^ in paired-end mode. Post-mapping quality control was done by Picard CalculateHsMetrics. Sequencing targets are considered all exons in RefSeq release 70. Genomic locations of the baits are defined in the specification of the SeqCap EZ v3.0 documentation.

### Gender tests for sample quality control

Based on the number of reads mapped to chromosome Y relative to all mapped reads, gender estimations were made. Tumor T53 was obtained from a female patient according to the patient information records but based on WES data its gender was estimated to be male (Supplementary Fig. 2). This observation questions the identity of the T53 sample, and therefore it cannot be guaranteed that the correct germ line sample was included. Therefore T53 was discarded in all further analyses.

### Somatic and pathogenic SNVs/INDELs

For variant calling, GATK^54^ was used to recalibrate base call scores, to re-align reads around INDELs and to call variants using the haplotype caller. Variants with low coverage (depth <5 reads), low GATK variant quality (GATK variant QUAL <50) and/or strand bias (FisherStrandBias >60) were discarded and remaining variants were annotated with ANNOVAR^55^. According to the rules of the local medical and ethical committee, genome-wide variants of germ line samples were anonymized and were merged into a single retinoblastoma germ line variant database. To identify somatic variants, only tumor variants that were not recorded in the retinoblastoma germ line variant data base were considered if they were at least covered by 10 reads. Two variants were considered identical when they had similar genomic coordinates, reference and mutant allele sequences, and the same GATK genotype calls (AA, AB, BB). To filter for somatic pathogenic variants, the somatic variants were required to be infrequent (<0.1%) in Exome Sequencing Project version 6500 and 1000 genomes 2014Oct. Variants were also were required to be marked “PASS” by GATK and were truncating (stop-gain/loss, frameshift-gain/loss, and INDELs) or splice site mutations or amino acid substitutions that were scored pathogenic by at least 2 out of 4 pathogenicity predicting programs (SIFT, LJB_LRT_Pred, PolyPhen2, and MutationTaster).

### Validation of *RB1* mutations by conventional DNA diagnostics

Validation of somatic and possibly pathogenic mutations was done by comparing *RB1* mutations identified by WES with results from diagnostics testing on tumor and germ line DNA by conventional methods described previously^56^. Germ line *RB1* genetic testing was performed for all patients but one (T29, bilateral patient). Tumor DNA diagnostics was available for 36 patients. 12/35 patients for whom no tumor DNA diagnostics was performed, a germ line *RB1* mutation was found. The remaining 23 patients without detectable germ line mutation and no available DNA diagnostics were all non-heritable unilateral patients diagnosed before 2007.

### Somatic copy number alterations

For somatic copy number alteration detection, the depth of coverage for 20 Kbps genomic windows was summarized using off-target reads only. The percentage of off-target reads per sample was on average 18% (range 11.9–23.4%, N = 141). Genomic windows that are hard to quantify with NGS (low mappability regions) according to the uniqueness of the ENCODE reference genome were discarded. Depth of coverage was normalized for GC content and mappability and log2-transformed ratios of tumor and matching germ line (Log2-R-ratios, LRR) were used for segmentation with DNAcopy under default parameters. These analyses were implemented by CopywriteR^10^.

### Allelic imbalance and loss of heterozygosity

All variants detected by GATK for all samples (N = 143) were pooled in a single database using VCFtools^57^. For each unique variant position and each sample, the B/variant allele frequency (BAF or VAF) and genotypes were calculated by SAMtools mpileup. Variant positions with at least 20x depth of coverage in at least 80% of the samples were considered, adding up to 208,822 unique markers. Using BAFsegmentation^58^, mirrored BAF (mBAF) were computed and mBAF segmentation was performed using allelic imbalance threshold 0.6 and a minimum of 5 consecutive markers for calling. Allelic imbalance was called loss of heterozygosity when segmented mBAF values exceeded 0.8.

## Additional Information

**Data availability**: Sequencing data is stored at the European Genomics Archive under accession number EGAS00001001690 and access is controlled by Data Access Committee EGAC00001000431.

**How to cite this article**: Kooi, I. E. *et al.* Somatic genomic alterations in retinoblastoma beyond RB1 are rare and limited to copy number changes. *Sci. Rep.* **6**, 25264; doi: 10.1038/srep25264 (2016).

## Supplementary Material

Supplementary Information

Supplementary Dataset 1

Supplementary Dataset 2

Supplementary Dataset 3

Supplementary Dataset 4

Supplementary Dataset 5

Supplementary Dataset 6

Supplementary Dataset 7

## Figures and Tables

**Table 1:** Somatic and possibly pathogenic variants for genes that showed at least 2 variants, excluding *RB1.* DP = depth of coverage, VAF = Variant allele frequency.

Gene	Type	Refseq cDNA annotation	DP	VAF	ID
*BCOR*	stopgain SNV	NM_001123384:c.T4472A:p.L1491X	36	0.92	T66
*BCOR*	nonsynonymous SNV	NM_001123383:c.G3001C:p.E1001Q	71	0.46	T63
*BCOR*	frameshift deletion	NM_001123384:c.3314delA:p.D1105fs	82	0.50	T61
*BCOR*	stopgain SNV	NM_001123383:c.C2926T:p.R976X	56	0.38	T57
*BCOR*	frameshift deletion	NM_001123384:c.4047_4053del:p.1349_1351del	48	0.19	T23
*CREBBP*	nonsynonymous SNV	NM_001079846:c.T4308G:p.C1436W	81	0.17	T62
*CREBBP*	nonframeshift deletion	NM_001079846:c.6629_6631del:p.2210_2211del	11	0.27	T47
