# Supplementary material for: Somatic genomic alterations in retinoblastoma beyond RB1 are rare and limited to copy number changes
Source: Sci Rep. 2016 Apr 29;6:25264. doi: 10.1038/srep25264 (PMC4850475; doi:10.1038/srep25264)
Supplement: Supplementary Information [file srep25264-s1.pdf]

Somatic genomic alterations in retinoblastoma beyond *RB1* are rare and limited to copy number changes

Irsan E. Kooi[a], Berber M. Mol[a], Maarten P.G. Massink[b], Najim Ameziane[a], Hanne Meijers-Heijboer[a], Charlotte J. Dommering[a], Saskia E. van Mil[a], Yne de Vries[a], Annemarie H. van der Hout[c], Gertjan J.L. Kaspers[d], Annette C. Moll[e], Hein te Riele[a,f], Jacqueline Cloos[c,g], Josephine C. Dorsman[a]

[a]*Department of Clinical Genetics, VU University Medical Center, Van der Boechorststraat 7, 1081BT, Amsterdam, The Netherlands; ei.kooi@vumc.nl, b.mol@vumc.nl,, n.ameziane@vumc.nl, h.meijers-heijboer@vumc.nl, cj.dommering@vumc.nl, se.vanmil@vumc.nl, y.waterham@vumc.nl, jc.dorsman@vumc.nl*

[b]*Department of Medical Genetics, Center for Molecular Medicine, University Medical Center Utrecht, Universiteitsweg 100, 3508 AB, Utrecht, The Netherlands; M.P.G.Massink@umcutrecht.nl*

[c]*Department of Genetics, University Medical Centre Groningen, University of Groningen, 9700 RB, Groningen, The Netherlands; a.h.van.der.hout@medgen.umcg.nl*

[d]*Department of Pediatric Oncology/Hematology, VU University Medical Center, De Boelelaan 1117, 1081 HV, Amsterdam, The Netherlands; gjl.kaspers@vumc.nl*

[e]*Department of Ophthalmology, VU University Medical Center, de Boelelaan 1117, 1007 MB Amsterdam, the Netherlands; a.moll@vumc.nl*

[f]*Division of Biological Stress Response, Netherlands Cancer Institute, Plesmanlaan 121, 1066 CX, Amsterdam, The Netherlands.; h.t.riele@nki.nl*

[g]*Department of Hematology, VU University Medical Center, de Boelelaan 1117, 1081 HV, Amsterdam, The Netherlands.; j.cloos@vumc.nl*

*Correspondence to: jc.dorsman@vumc.nl, J-376, Van der Boechorststraat 7, 1081BT Amsterdam, +31-20-4448424

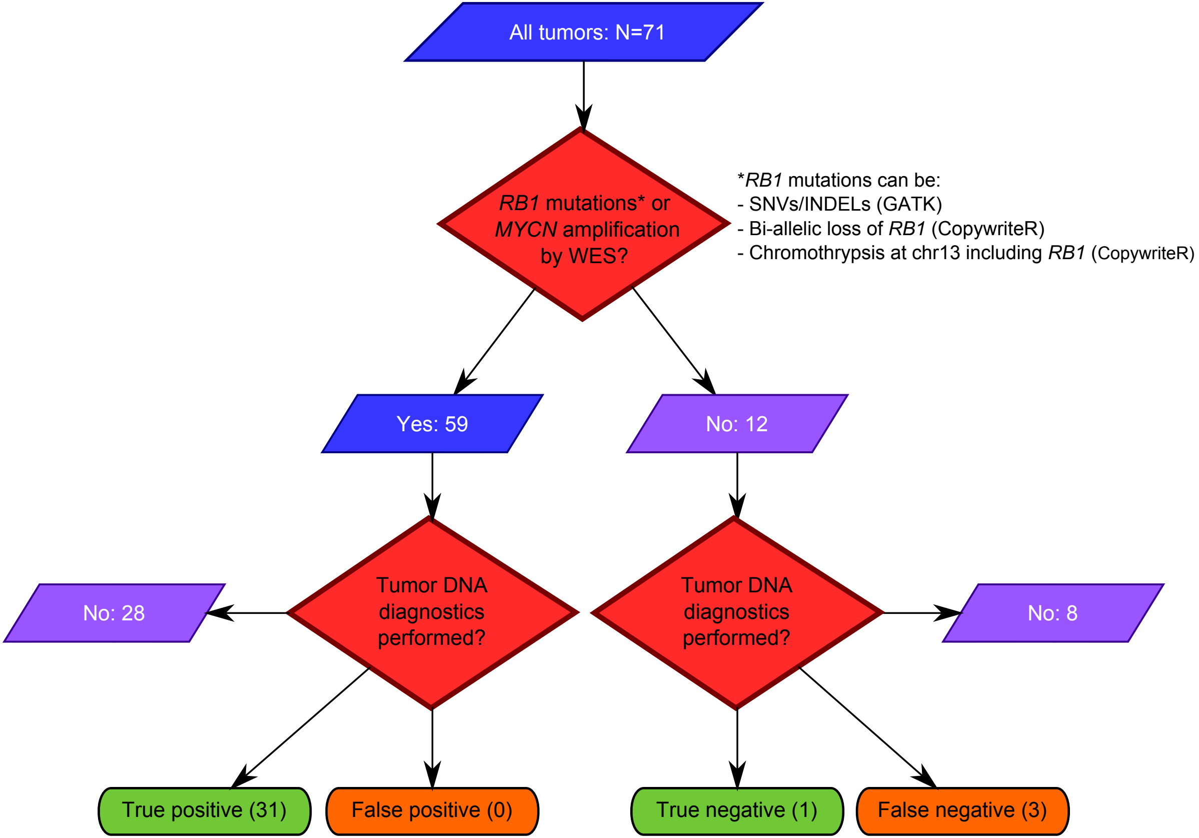
All tumors: N=71
*RB1* mutations* or *MYCN* amplification by WES?
**RB1* mutations can be:
- SNVs/INDELs (GATK)
- Bi-allelic loss of *RB1* (CopywriteR)
- Chromothrypsis at chr13 including *RB1* (CopywriteR)
Yes: 59
No: 12
Tumor DNA diagnostics performed?
Tumor DNA diagnostics performed?
No: 28
No: 8
True positive (31)
False positive (0)
True negative (1)
False negative (3)

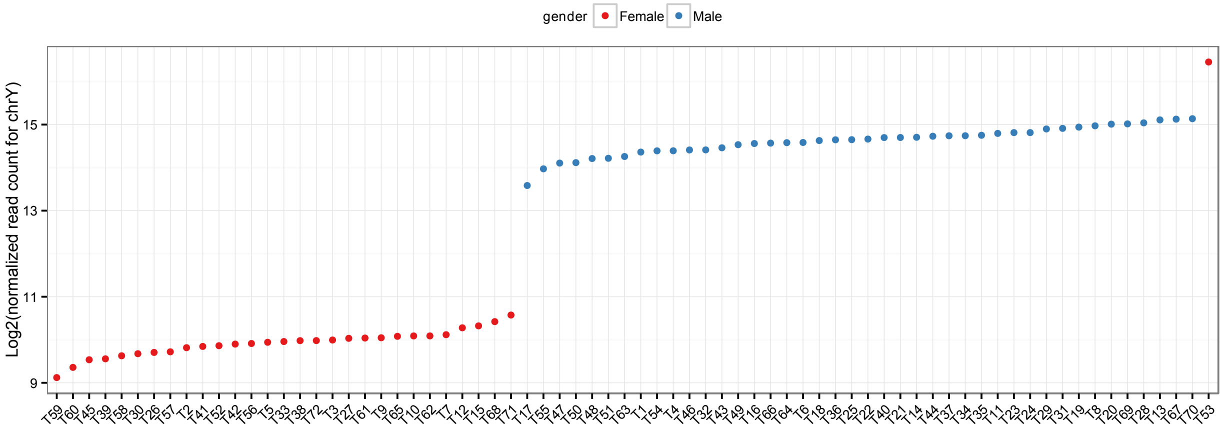
gender
Female
Male
Log2(normalized read count for chrY)
9
11
13
15
T59 T60 T45 T39 T58 T30 T26 T57 T2 T41 T52 T42 T56 T5 T33 T38 T72 T3 T27 T61 T9 T65 T10 T62 T7 T12 T15 T68 T71 T17 T55 T47 T50 T48 T51 T63 T1 T54 T4 T46 T32 T43 T49 T16 T66 T64 T6 T18 T36 T25 T22 T40 T21 T14 T44 T37 T34 T35 T11 T23 T24 T29 T31 T19 T8 T20 T69 T28 T13 T67 T70 T53

**Supplementary figure 1: Assessment of WES performance by comparing RB1 variants with DNA diagnostics results.** Inactivation of RB1 (SNVs/INDELs, SCNAs or 13q chromothrypsis) or MYCN amplification was observed for 59/71 (83%) of the tumors (WES positives). For 31/59 (55%) WES positives conventional tumor DNA diagnostics was performed to aid determination of retinoblastoma heredity and confirmed WES results (true positives). For 12/71 (17%) tumors no RB1 inactivation or MYCN amplification could be found (WES negatives). For 4/12 WES negatives tumor DNA diagnostics was performed showing that for one WES negative no alterations could be found (true negative) and for the three other WES negatives, RB1 mutations were missed (false negatives).

**Supplementary figure 2: Quality control by gender estimation based on WES data.** For each tumor (dots) the number of reads mapping to chromosome Y normalized for total number of mapped reads was log2-transformed and plotted. Colors indicate the gender according to patient records (red: female, blue: male). Sample T53 was the only sample for which gender estimation based on WES data was inconsistent with patient records and was therefore omitted from further analyses.
